# Wound Healing Activity and Mechanisms of Action of an Antibacterial Protein from the Venom of the Eastern Diamondback Rattlesnake (*Crotalus adamanteus)*

**DOI:** 10.1371/journal.pone.0080199

**Published:** 2014-02-14

**Authors:** Ramar Perumal Samy, Matheswaran Kandasamy, Ponnampalam Gopalakrishnakone, Bradley G. Stiles, Edward G. Rowan, David Becker, Muthu K. Shanmugam, Gautam Sethi, Vincent T. K. Chow

**Affiliations:** 1 Venom and Toxin Research Programme, Department of Anatomy, Yong Loo Lin School of Medicine, National University of Singapore, Singapore, Singapore; 2 Infectious Diseases Programme, Department of Microbiology, Yong Loo Lin School of Medicine, National University of Singapore, Singapore, Singapore; 3 Infection & Immunity Programme, Singapore Institute for Clinical Sciences, Agency for Science, Technology and Research, Brenner Centre for Molecular Medicine, Singapore, Singapore; 4 Integrated Toxicology Division, US Army Medical Research Institute of Infectious Diseases, Fort Detrick, Maryland, United States of America; 5 Strathclyde Institute of Pharmacy and Biomedical Sciences, University of Strathclyde, Glasgow, United Kingdom; 6 Department of Anatomy and Developmental Biology, University College London, London, United Kingdom; 7 Department of Pharmacology, Clinical Research Centre, Yong Loo Lin School of Medicine, National University of Singapore, Singapore, Singapore; Charité-University Medicine Berlin, Germany

## Abstract

Basic phospholipase A_2_ was identified from the venom of the eastern diamondback rattlesnake. The *Crotalus adamanteus* toxin-II (CaTx-II) induced bactericidal effects (7.8 µg/ml) on *Staphylococcus aureus,* while on *Burkholderia pseudomallei* (KHW), and *Enterobacter aerogenes* were killed at 15.6 µg/ml. CaTx-II caused pore formation and membrane damaging effects on the bacterial cell wall. CaTx-II was not cytotoxic on lung (MRC-5), skin fibroblast (HEPK) cells and in mice. CaTx-II-treated mice showed significant wound closure and complete healing by 16 days as compared to untreated controls (**P<0.01). Histological examination revealed enhanced collagen synthesis and neovascularization after treatment with CaTx-II versus 2% Fusidic Acid ointment (FAO) treated controls. Measurement of tissue cytokines revealed that interleukin-1 beta (IL-1β) expression in CaTx-II treated mice was significantly suppressed versus untreated controls. In contrast, cytokines involved in wound healing and cell migration i.e., monocyte chemotactic protein-1 (MCP-1), fibroblast growth factor-basic (FGF-b), chemokine (KC), granulocyte-macrophage colony-stimulating factor (GM-CSF) were significantly enhanced in CaTx-II treated mice, but not in the controls. CaTx-II also modulated nuclear factor-kappa B (NF-κB) activation during skin wound healing. The CaTx-II protein highlights distinct snake proteins as a potential source of novel antimicrobial agents with significant therapeutic application for bacterial skin infections.

## Introduction

Antimicrobial proteins and peptides play a major role in innate immunity by interacting directly with bacteria and killing them [1]. These multifunctional proteins have wound healing activity and receptor-mediated effects on eukaryotic cells [2]. Previously reported mammalian proteins and peptides such as defensins [3], cathelicidins [4], and peptide LL-37 [5] can cause hemolysis of human erythrocytes and are cytotoxic at higher doses that might adversely affect wound healing [6], [7]. In addition, emergences of multi-drug resistant (MDR) bacteria generated by specific mutations on antibiotic targets, and acquired by transposon genes [8], are one of the important healthcare concerns for long-term management. Antibiotics used for treatment of *Staphylococcus aureus* infected surgical wounds and other skin related infections include methicillin [9], vancomycin [10], glycopeptides [11], and beta-lactams [12]. Following *S. aureus* infection, wound healing after treatment with some of these antibiotics has been reported to be only moderately successful in many cases [13].

However, the prevalence of bacterial resistance to conventional antibiotics has prompted an intensive search for new therapeutic agents including various antimicrobial peptides of animal origin [14]. Proteins/peptides with potent antimicrobial activity have been found in a wide variety of organisms, including snakes. Their venom, particularly crotalidae venoms [15], is a rich natural source for discovery and development of novel antimicrobial agents. Crotapotin, a secretory phospholipase A_2_ isolated from the venom of *Crotalus durissus terrificus*, shows antibacterial activity [16] as well as antiviral activity against the human immunodeficiency virus [17], [18]. Crotamine is a small basic myotoxin from South American rattlesnake (*Crotalus durissus terrificus*) killed via membrane permeabilization of *Escherichia coli* with the MICs ranging from 25–100 µg/ml at 1–2 h and fail to show any hemolytic activity to erythrocytes. Crotamine antimicrobial peptides (AMP) structure similar to that many class of beta-defensin [19]. Acidic PLA_2_ [20], both Asp49 and Lys49 PLA_2_ homologues [21], have previously been shown to possess bactericidal activity [22]. A cationic protein isolated from venom of the inland taipan (*Oxyuranus microlepidotus)* selectively and dose-dependently kills Gram-positive bacteria through membrane disruption [23]. These molecules are very attractive because of their biochemical diversity, broad spectrum of activity against enveloped viruses, bacteria, fungi, protozoa, parasites, and promotion of wound healing [25], [26]. The eastern diamondback rattlesnake (*Crotalus adamanteus)* belongs to the family Viperidae (subfamily Crotalinae) and is widely distributed in the United States [27]. Many venom enzymes from various species, including PLA_2_ **,** *hyaluronidase*, L-amino acid oxidase, metalloproteinases, as well as myoprotein-CAM-protein and hemorrhagic protein possess potent pharmacological effects such as myotoxic, neurotoxic and edematogenic activities [26]. However, relatively very little is known regarding the antimicrobial activity of *C. adamanteus* venom [15] or its components. The aim of the present finding was to evaluate the antimicrobial and wound healing activity of a *C. adamanteus* venom protein (CaTx-II) in a mouse model for *S. aureus* wounds.

## Materials and Methods

### Venom

Required quantities (10 g) of the crude venom of *Crotalus adamanteus* (Viperidae: subfamily Crotalinae) were purchased from Venom Supplies Pte Ltd, Tanuda, Southern Australia, and stored desiccated at 4°C in individual containers at the Department of Anatomy, NUS, Singapore.

### Purification of Protein (CaTx-II)

*Crotalus adamanteus* venom (1 g) was dissolved completely in 10 ml of 1 M Tris-HCl (pH 7.4), and after centrifugation at 1800×g for 15 min, the clear yellowish solution was sterilized by filtration through a 0.22 µm membrane filter (Nalge Nunc International, Rochester, New York, USA). Venom samples (1 ml) diluted in 9 ml of Tris-HCl was then injected into a Superdex G-75 column attached to a high performance liquid chromatography system (HPLC, AKTA GE Healthcare, Denmark). Eluents (1 ml fractions) were monitored at 280 nm and collected in 1.5 ml eppendorf tubes. All the fractions were tested for enzymatic and antibacterial effects, and the fraction that showed the highest enzymatic and antibacterial effects were then subjected to reverse-phased liquid chromatography (RP-HPLC) via a C18 column (4.6×150 mm, Phenomenex, Upsala, Sweden), using a linear gradient of aqueous acetonitrile (80%) in 0.1% trifluoroacetic acid (Sigma Aldrich Co, St Louis, Missouri, USA) at a flow rate of 1.0 ml per min. Subsequent purification of active fractions were derived by using a C8 RP-HPLC column (100 Å, 4.6×250 mm) [1].

### Sodium Dodecyl Sulphate Polyacrylamide Gel Electrophoresis (SDS-PAGE)

*C. adamantetus* RP-HPLC fractions, and the final purified fraction of protein (CaTx-II), were analyzed by SDS-PAGE according to Laemmli [28]. The molecular weights of protein bands were determined using standard markers (Bio-Rad, California, USA). Protein concentrations were determined by Lowry’s method [29].

### Phospholipase A_2_ (PLA_2_) Activity

The PLA_2_ activity of *Crotalus adamanteus* venom, fractions, and purified proteins were determined as described [1], with slight modifications. Each of the above sample solutions (containing 20 µg protein concentration) of 180 µl was added to the substrate solution containing 5 mM Triton X 100, 5 mM phosphatidylcholine (Sigma Co, St. Louis, Missouri, USA), 2 mM HEPES, 10 mM calcium chloride (Merck & Co., Inc, New Jersey, USA) plus 0.124% bromothymol blue dye (wt/vol) in water (pH 7.5). After incubating for 10 min at 37°C, absorbance was determined at 590 nm in a microtiter spectrophotometer (Research Instrument, Virginia, USA). Results were expressed in nmoles of acid per minute per µg of venom protein.

### Mass Spectrometry, Amino Acid Composition, and Sequencing Analyses

The molecular mass of CaTx-II was analyzed using matrix-assisted laser desorption time of flight mass spectrometry (MALDI-TOF/MS) in positive ionization mode. Alpha-cyano-4-hydroxycinnamic acid used to perform with a Voyager-DE STR (Applied biosystems, Farmingham, Massachusetts, USA). Amino acid composition and sequencing analyses were performed as described previously [1]. The terminal sequence of CaTx-II samples were performed at the Protein and Proteomic Center, Department of Biological Science, NUS, Singapore using a fully automated ABI Procise 494 protein sequencer (Applied Biosystem,) system that employs Edman’s degradation reaction for sequential separation of N-terminal amino acids.

### *In vitro* Antimicrobial Assays

Bacterial cultures of Burkholderia pseudomallei (TES), Burkholderia pseudomallei (KHW), Escherichia coli, Enterobacter aerogenes, Klebsiella pneumoniae, Streptococcus pneumoniae, Proteus vulgaris, Proteus mirabilis, Pseudomonas aeruginosa, and Staphylococcus aureus were obtained from the Department of Microbiology, NUS, Singapore. Venom fractions and purified CaTx-II were tested for antimicrobial activity. Bacteria were grown in Mueller Hinton (MH) and Tryptic Soya (TS) broth (Oxoid Limited, Basingstoke, Hampshire, UK) to exponential phase (0.5–1.5×10^6^ colony forming units (CFU per ml)) with an A600 of 0.8, representing 12,000 cfu/ml. 20 ml of MH or TS agar medium was poured into each petri dish (140 mm×20 mm) obtained from Sterilin Limited, UK. After solidification, bacteria were swabbed uniformly onto the agar surface by a sterile cotton swab. 20 µl of each test sample (250 µg/ml) was applied to a sterile blank disc (6 millimeter in diameter, BD Bioscience, New Jersey, USA) placed on the surface of the MH and TS agar medium, and incubated at 37°C for 24 h. The blank disc containing sterile water (20 µl) served as a negative control, while the commercially available antibiotic discs of chloramphenicol (30 µg/disc) and streptomycin (30 µg/disc) served as positive controls. Three replications were used for each assay and effects were recorded as a clear zone of inhibition, measured diameter in mm, after incubation [1], [30].

### *In vitro* Bactericidal Activities of CaTx-II

Minimum Inhibitory Concentrations (MICs) of CaTx-II against different bacteria were determined by the MH and TS broth dilution method [31]. A single bacterial colony was used to inoculate each MH and TS broth (5 ml/tube) subsequently incubated at 37°C for 6–8 h (exponential-phase cells) before adjusting with sterile medium to a 0.5 McFarland turbidity standard. The adjusted bacterial cultures were diluted to approximately 10^5^–10^6 ^CFU/ml before using in the MIC assays. Bacteria (50 µl) were introduced into each well (containing 100 µl of medium) of a sterile 96-well plate, followed by addition of another 50 µl of serially diluted CaTx-II (250, 125, 62.5, 31.25, 15.6, 7.8 and 3.9 µg/ml). 100 µl of medium containing bacteria served as a control, and 100 µl of medium alone served as a blank. Plates were incubated for 24 h at 37°C, and inhibition of bacterial growth was determined by measuring turbidity at 560 nm. Each MIC was determined from three independent experiments. The lowest concentrations required for inhibiting the bacterial growth (MICs) were calculated by statistical analysis [32]. Minimum bactericidal concentrations (MBCs) were determined as described by Perumal Samy et al, [1]. Briefly, log-phase bacteria were obtained from MH and TS broth cultures, and their concentration adjusted to 10^5^–10^6^ CFU/ml. Bacterial suspensions (50 µl) were incubated at 37°C for 24 h with 50 µl of CaTx-II (250–3.9 µg/ml) and 100 µl of MH and TS broth in a 96-well plate. To assess bacterial viability, these broth cultures were plated on MH and TS agar and counted following 24 h of growth at 37°C. The percentage of bactericidal effects was calculated versus bacterial control.

### Scanning Electron Microscope (SEM)

Bacterial cells were grown in MH and TS broth (5 ml per tube) to an exponential phase. Approximately 2×10^6^ CFU/ml were then centrifuged at 12,000×g for 10 min at 4°C, washed twice with 2 ml of 10 mM sodium phosphate buffer, and resuspended in 2 ml of the same buffer. The bacteria were incubated for 30 min with different concentrations of CaTx-II, depending on their pre-determined MIC values for each strain (i.e., 7.8 µg/ml for *S. aureus* and *B. pseudomallei* (KHW), 15.6 µg/ml for *B. pseudomallei* (TES) and 31.25 µg/ml for *E. aerogenes*, *P. vulgaris*, and 62.5 µg/ml for *P. mirabilis*. After incubation, each sample was spread onto a poly L-lysine (Sigma Aldrich, St Louis, Missouri, USA) coated glass slide to immobilize bacteria at 37°C for 24 h. The bacteria were fixed with 2.5% glutaraldehyde in 0.1 M sodium phosphate buffer, extensively washed with the same buffer, and dehydrated with an ethanol gradient (50–100%). After critical-point drying and gold coating, the samples were observed with a Philips XL 30 microscope [1].

### Cell Culture

Human lung (MRC-5) and skin fibroblast (HEPK) cells were purchased from the American Type Culture Collection (Virginia, USA), and the cell proliferation Kit II was from Roche Applied Sciences, Indianapolis, Indiana, USA. Sterile Roswell Park Memorial Institute (RPMI) medium, fetal bovine serum (FBS), 10 mM phosphate buffered saline (PBS, pH 7.4), and 10 mM HEPES were purchased from the National University Medical Institute (NUMI), Singapore. All chemicals were of analytical and cell culture grade.

### *In vitro* Cytotoxicity

The MRC-5 and HEPK cells were cultured in 72 cm^2^ flasks to a density of 10^6^ cells per flask in RPMI as well as Dulbecco's Modified Eagle Medium (DMEM) culture medium, supplemented with 10% FBS and 1 ml of HEPES. Cell viability was measured using tetrazolium salts (XTT) as described previously [33]. Briefly, the cells were allowed to adhere to the flask bottom overnight at 37°C in a humidified atmosphere of 5% CO_2_ and 95% air. Cells were split and reseeded into new flasks, etc. To analyze the cytotoxic effects, CaTx-II was applied to cultured fibroblasts tested at different concentrations (15.6–2000 µg/ml) in a 96 well plate. The assay plates were incubated at 24 and 48 h time intervals. Cell proliferation was spectrometrically quantified using an ELISA plate reader at 490 nm (Bio-Rad, Hercules, California, USA). All assays were performed in triplicate (n = 3).

### Cytolysis Determination by Lactate Dehydrogenase (LDH) Release

The cytotoxicity of CaTx-II upon human cells (lung and skin fibroblasts) was evaluated by measuring LDH release using a cytotoxicity detection kit (Roche Mannheim, Germany). Cells (10^6^) were cultured in 96-well micro titer plates with RPMI and DMEM medium (NUMI, Singapore) supplemented with 10% (vol/vol) FBS and 1 ml of penicillin/streptomycin (1 x). CaTx-II was added into three wells per dose (15.6–2000 µg/ml), while cells without any treatment served as a control. The cells were further incubated at 37°C in the presence of 5% CO_2_ for 24 and 48 h. After incubation, the LDH released into the culture medium from each well was collected following centrifugation at 10,000×g, 4°C for 5 min. A 200 µl aliquot was used for the quantification of cell death and lysis, based on LDH released from the cytosol of damaged cells into the supernatant. The assay was performed in triplicate [34].

### Mice

Pathogen free, 8–12 weeks old male Swiss Albino mice (body weight 25–30 gm) were obtained from the Animal Breeding Center, Sembawang, Singapore. The animals were maintained for 12 h under light/dark conditions, and were given appropriate diet plus water *ad libitum*. All experimental animals were approved by the ethics committee, NUS, Singapore (Approval number 055/2007).

### *In vivo* Toxicity (Mice)

The toxic effect of purified CaTx-II was studied using a modified method previously reported [1]. The toxic effect of rattlesnake venom and CaTx-II was assessed in Swiss Albino male and female mice (1–14 µg/kg, body weight) by intraperitoneal (i.p.) injection in 200 µl of PBS. Six animals per dose were used, and the mortality recorded.

### Antibodies

Monoclonal or polyclonal antibodies (anti-mouse type I collagen and anti-rat CD68, Santa Cruz, California, USA) and secondary antibodies labeled with FITC (Sigma Co., St. Louis, Missouri, USA) or Cy3 (Santa Cruz, California, USA), and DAPI stain (Daiko, Japan). All other reagents were of analytical grade (Roche, Germany).

### Skin Wound Preparation and Macroscopic Observation

Mice (n = 6 per group) were anesthetized with meditorin and ketamin injections (i.p., 20 and 100 mg/kg body weight, respectively), and their skin hair from the back shaved with a sterile razor blade. After wiping the shaved area with 70% ethyl alcohol, an incision 2 cm (full thickness of the wound 4 mm×5 mm in length and width) was made. Fifty microliters of *S. aureus* (10^6^ cfu/ml) suspended in growth medium in sterile PBS were applied onto the wound region. Mice were separated into individual cages and kept for 2 days prior to topical application of CaTx-II (10 mg/kg, body weight), antibiotic (2% FAO) and untreated wound control (WCtrl). Wound healing of the animals was monitored daily by measuring the area of wound closure with a tracing paper at 0–16 days. The wound healing was then analyzed and the percent reduction of original wound area calculated. Animals were killed after 16 days by excess CO_2_ inhalation, and the skin biopsy samples collected from the bisected center from the skin wound for biochemical studies [35]. For analysis of collagen and pro-inflammatory cytokines, wound tissues were frozen immediately in liquid nitrogen and stored at −80°C until assay time.

### Immunohistochemistry

For H & E staining, sections of skin were fixed in 4% paraformaldehyde in PBS for 16 h before embedding into paraffin. Thin sections (7 µm ) were cut and processed in an alcohol series (50–100%) for 5 min, stained with H & E for 3 min, and then rinsed in tap water for 3 min, before mounting on glass slides with an embedding media of Histo-Clear (Merck & Co, New Jersey, USA). The sections were examined under the microscope (Carl Zeiss Inc., Microimaging, LLC, Thornwood, New York, USA). Masson’s Trichrome staining was performed for identification of collagen [36]. For immunohistochemical staining, excess tissues were trimmed down and 7 µm thick paraffin sections were used to detect different antigens. Type I collagen deposits were immunodetected according to the described protocol. Tissue sections were incubated overnight with the primary antibodies (1∶50 or 1∶100 dilutions for collagen, and 1∶200 for CD-68) at 4°C, washed three times in PBS, and then incubated with peroxidase-conjugated secondary antibodies (1∶1000 in blocking solution) for 5 min. Sections were counterstained with 4, 6-diamidino-2-phenylindole (DAPI) (Invitrogen, Carlsbad, California, USA) and stored at 4°C. The sections were examined by a Laser Confocal microscope (Olympus Imaging America Inc., Center Valley, Pennsylvania, USA) connected to software (Flow view, FV10.AS), version (2.0 viewer).

### Measurement of Collagen in Wound Tissues

Collagen content of skin wounds were determined by an ELISA using an assay kit for mouse type I collagen (Chondrex, Inc., Redmond, Washington, USA) according to the manufacturer’s instructions. Briefly, the microtiter plate was washed 3 times and blocked with 100 µl of blocking buffer solution A for 1 h at room temperature. Skin tissues (100 mg) were cut into small pieces by sterile razor blade, each skin sample was homogenized in 0.5 ml of SDS-buffer, (pH 7.6) by a variable speed tissue homogenizer (Model 7000, Eberbach Corporation, Michigan, USA). Homogenate was centrifuged (10,000×g, at 4°C for 5 min) to remove the supernatant containing the soluble protein and diluted in 1∶1000 µg/ml of solution B. Sample, standard, or blank solution B (100 µl) was added to the wells in triplicate and incubated over night at 4°C. After 24 h, the plates were washed 3 times with 1×wash buffer, and the secondary antibody (100 µl) introduced into each well. Following incubation at room temperature for 2 h, the plates were washed 3 times with wash buffer, incubated with OPD solution (100 µl/well) for 30 min at room temperature, and 50 µl of sulfuric acid (50 mM) added to stop the reaction before reading the OD at 490 nm.

### Extraction of Wound Samples

Skin wound tissues were excised by using a sterile razor blade to include the surrounding wound tissues (100 mg weight of tissue taken per animal) 16 days after treatment. The samples were frozen in liquid nitrogen and stored at −80°C until analysis. The tissue samples were homogenized in tissue protein extraction reagent (T-PER containing 25 mM bicine and 150 mM sodium chloride, pH 7.6, including protease inhibitor cocktail purchased from Thermo Fisher Scientific (Rockford, Illinois, USA) and stored at −80°C prior to analysis. After dilution of the homogenates with MH and TS broth, 20 µl of each sample was swabbed on the MH and TS agar plates and incubated at 37°C for 24 h. Bacterial colonies were counted and CFU/ml calculated for the different treatment groups.

### Analysis of Proinflammatory Cytokines

Skin wound tissues were collected and extracted by standard protocols. Total protein of the skin homogenates were determined by using a Bio-Rad Protein Assay (Bio-Rad Laboratories, Hercules, California, USA). The concentrations of proinflammatory cytokines interleukin-1 beta (IL-1β), IL-6, IL-10, chemokine (KC), tumor necrosis factor-alpha (TNF-alpha), fibroblast growth factor-basic (FGF-b), granulocyte-macrophage colony-stimulating factor (GM-CSF), monocyte chemotactic protein-1 (MCP-1) and macrophage inflammatory protein-1 beta (MIP-1β) levels were measured by using a commercially available multiplex cytokine kit (Mouse Inflammation Kits, BD Pharmingen, San Diego, California, USA) according to the manufacturer’s instructions.

### Western Blot Analysis

Vascular endothelial growth factor (VEGF) antibody was obtained from Santa Cruz Biotechnology (Santa Cruz, CA, USA). Phospho-specific anti-p65 (Ser 536) was purchased from Cell Signaling (Beverly, MA, USA). β-actin antibody were obtained from Sigma Chemicals Co (St. Louis, MO, USA). To determine the levels of various proteins, skin wound tissue extracts were prepared and fractionated by SDS-polyacrylamide gel electrophoresis (PAGE) as described previously [37]. After electrophoresis, the proteins were electro-transferred to a nitrocellulose membrane, blocked with 5% nonfat milk to minimize non-specific binding, and probed with various antibodies (1∶1000) overnight at 4°C. The blot was washed, exposed to HRP-conjugated secondary antibodies for 2 h, and the expression of various proteins was detected by chemiluminescence emission (ECL; GE Healthcare**,** Little Chalfont, Buckinghamshire, UK).

### Statistical Analysis

Each experimental group, six animals were used for the wound healing study (n = 6). All assays were performed in triplicate. Data are presented as mean ± SD (standard deviation). Statistical analysis was carried out by ANOVA for comparison of multiple groups, or by Student’s t-test for two-group comparisons. Levels of significance are indicated as *p<0.01, **p<0.05.

## Results

### Purification and Identification of Protein (CaTx- II) Molecules

**Figure 1:**
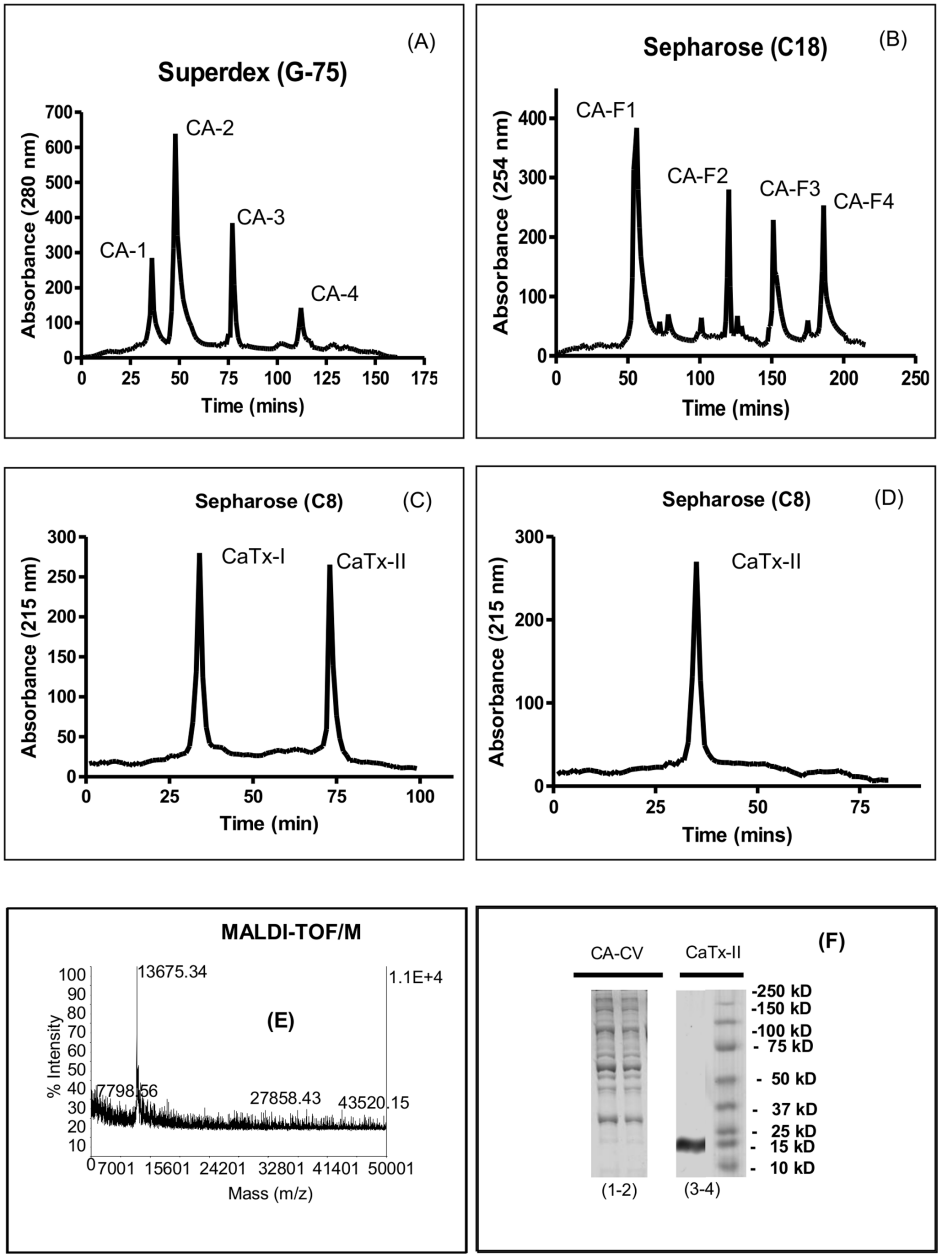
Purification of *Crotalus adamanteus* toxin-II (CaTx-II) from Eastern Diamondback Rattlesnake venom. (A) High Performance Liquid Chromatography (HPLC) profiles of *C. adamanteus* crude venom from a Superdex G-75 column, (B–D) Reverse-phase (RP)-HPLC chromatograms from Sepharose C18 and C8 columns, (E) Molecular mass of pure CaTx-II, (F) Sodium dodecyl sulphate-polyacrylamide gel electrophoresis (SDS-PAGE) profile of RP-HPLC fractions, lanes indicate: CA-CV *C. adamanteus* crude venom (1–2), lane (4) homogeneity of CaTx-II confirmed by SDS-PAGE as 15 kDa of CA-F1 - reverse-phase fraction and (5) marker, 25 µg of protein loaded per lane, respectively.

Purification of *C. adamanteus* venom by Superdex G-75 gel-filtration chromatography provided four (CA1–CA4) fractions (Fig.1 A). Upon fractionation of the enzymatically and antimicrobially most active fraction (CA2) by C18 RP-HPLC chromatography (Fig. 1B), it was further resolved into four fractions (CA-F1 to CA-F4). The antimicrobially-active fraction CA-F1 was further fractionated through a C8 column to obtain a purified protein identified as CaTx-II. Each fraction was purified in subsequent steps to homogeneity (Fig. 1 C–D). Preliminary mass analysis (MALDI-TOF/MS) revealed a 13675.34 kDa protein (Fig.1 E). SDS-PAGE (Fig.1 F) shows a single band with a molecular mass of 15 kDa. Elucidating PLA_2_ activity of column fractions showed variable levels of PLA_2_ activity, with the fraction containing CaTx-II displaying higher levels of PLA_2_ activity than that of other fractions or crude venom (Fig. S1 A–D). In addition, CaTx-II possessed the highest activity of any PLA_2_ purified from *C. adamanteus* venom.

### Amino Acid Analysis

The N-terminal amino acid sequence of CaTx-II is shown in table S1. When aligned and compared with the N-terminal sequences of other snake venom PLA_2_s, the sequence of CaTx-II is quite homologous with basic PLA_2_s isolated from other crotalidae venoms, with a sequence similarity of 70–80% found with the Lys-49 PLA_2_ sequence of crotapotin from *C. adamanteus* venom.

### Antimicrobial Activity of CaTx-II

When screened against a wide range of Gram-negative and Gram-positive bacteria using a disc-diffusion assay, CaTx-II showed very strong antimicrobial effect against *S. aureus,* B. pseudomallei (TES & KHW), E. aerogenes, P. vulgaris *and* P. mirabilis. (Fig. S2 A). It exerted maximum inhibition zones against *S. aureus* and *B. pseudomalleii* (KHW) compared to those of chloramphenicol or streptomycin (30 µg/disc). The most sensitive bacterium to CaTx-II was *S. aureus,* whereas against Gram-negative *B. pseudomallii* (strain KHW), CaTx-II was more active even at a lower dose of 20 µmole range than the antibiotics (30 µg). Similar effects were also observed with the less purified fraction (CA-F1) (Fig. S2 B). *At* higher concentrations, the crude venom showed more potent antimicrobial activity against the multi-drug resistant B. pseudomallei (KHW) than P. aeruginosa (Fig. S2 C). The CA-CV crude venom, CA-F1 isolated fractions, and the purified CaTx-II protein exhibited only moderate activity against *E. coli, P. aeruginosa*, *K. pneumoniae* and *S. pneumoniae* at the tested concentration. However, the growth inhibition curves clearly illustrated that CaTx-II was the most active antimicrobial agent against *S. aureus* than other tested fractions or control antibiotics (Fig. S2 D–E).

### Bacteriostatic (MIC) and Bactericidal (MBC) Assays

MICs indicated that CaTx-II had an interesting inhibitory (bacteriostatic) effect even at the lowest dilutions against *S. aureus* (MIC value of 7.8 µg/ml), *B. pseudomallei* strain KHW and *B. pseudomallei* strain TES (MIC value of 15.6 µg/ml). A higher MIC value (62.5 and 125 µg/ml) was recorded against *P. mirabilis, K. pneumoniae,* and *P. aerugionsa* versus *P. vulgaris* (31.25 µg/ml) in Fig. S3. Whereas, bactericidal effects of CaTx-II against *S. aureus*, and *B. pseudomallei* (MBC values of 7.8–15.6 µg/ml) were very powerful and much more potent on a mole basis versus standard antibiotics (Fig. S4).

### Membrane Damaging Effects of CaTx-II

**Figure 2:**
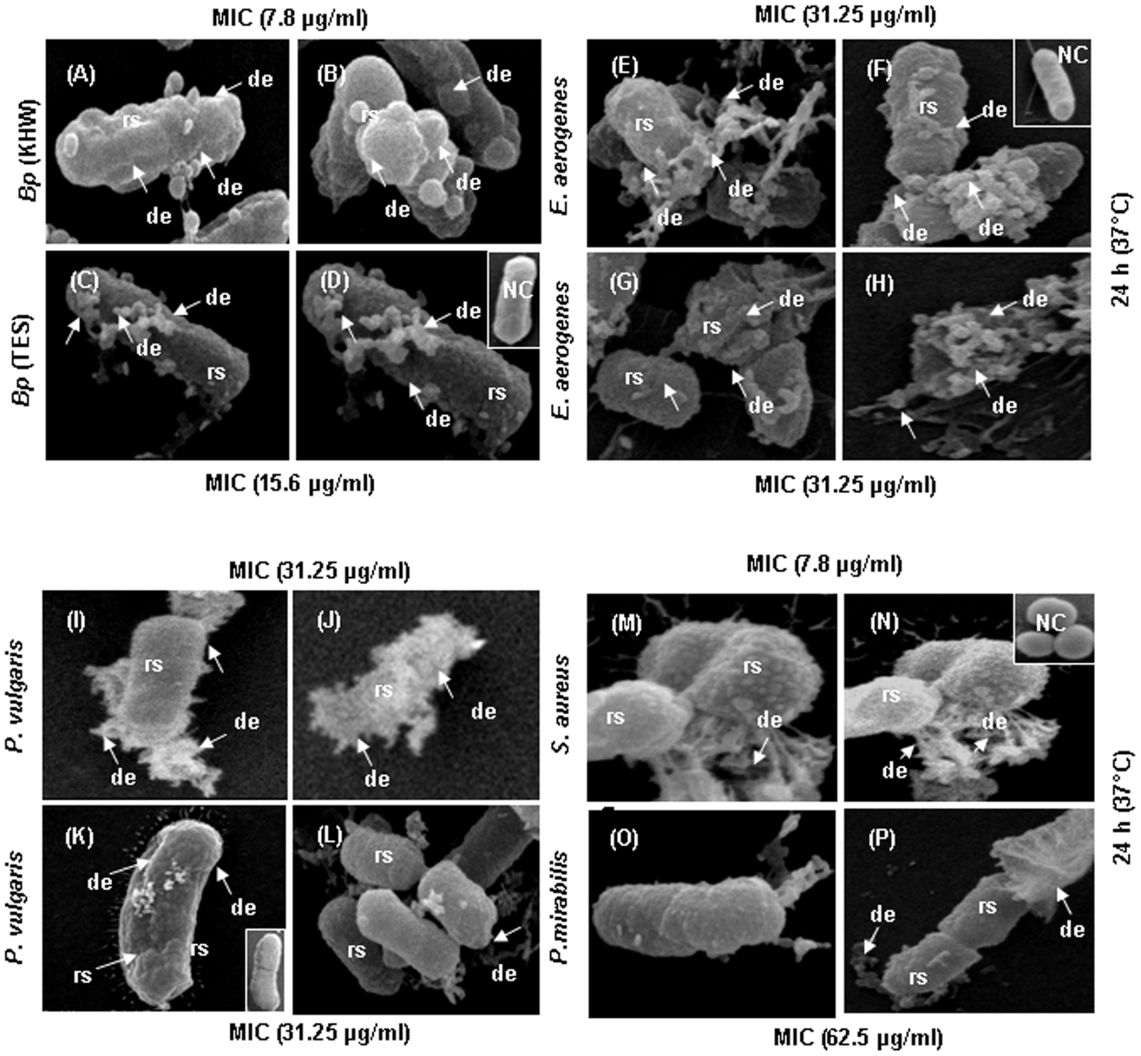
CaTx-II damages the bacterial surface as determined by SEM. (NC) Bacteria treated with PBS as a normal control. (A–D) Exposure to 7.8 and 15.6 µg/ml proteins induced mainly membrane-damaging effects upon *B. pseudomallei* (KHW &TES). (E–H) *E. aerogenes*, (I–L) *P. vulgaris,* (M–N) *S. aureus* incubated with 7.8 µg/ml of protein show a severely damaged cell membrane (disorganization), formation of membrane blebbing, and release of cellular content (cytoplasmic eruption). (O–P) *P. mirabilis* exposed to 62.5 µg/ml of CaTx-II have surface roughening and membrane damaging effects after 24 h. All samples were processed at 24 h for SEM analysis as described in the experimental procedures.

Further studies were done with CaTx-II to determine what, if any, overt effects might be seen on bacteria via SEM analysis. As observed on *B. pseudomallii* KHW (Fig. 2A–B), *B. pseudomallii* TES (Fig. 2C–D), *E. aerogenes* (Fig. 2E–H), *P. vulgaris* (Fig. 2I–L), *S. aureus* (Fig. 2M–N), and *P. mirabils* (Fig. 2O–P), the effects of this protein appeared quite rapidly (24 h) at the tested concentrations (3.9–250 µg/ml). CaTx-II was potent, even at the low concentrations, in killing the bacteria very efficiently through damaging the membrane. Pore formation and membrane-damaging effects of CaTx-II varied depending upon the bacterium. Although its main effect is on bacterial cell membranes as the primary target, it is unclear whether CaTx-II may also act on other intracellular targets.

### Cell Viability & Cytotoxicity

**Figure 3:**
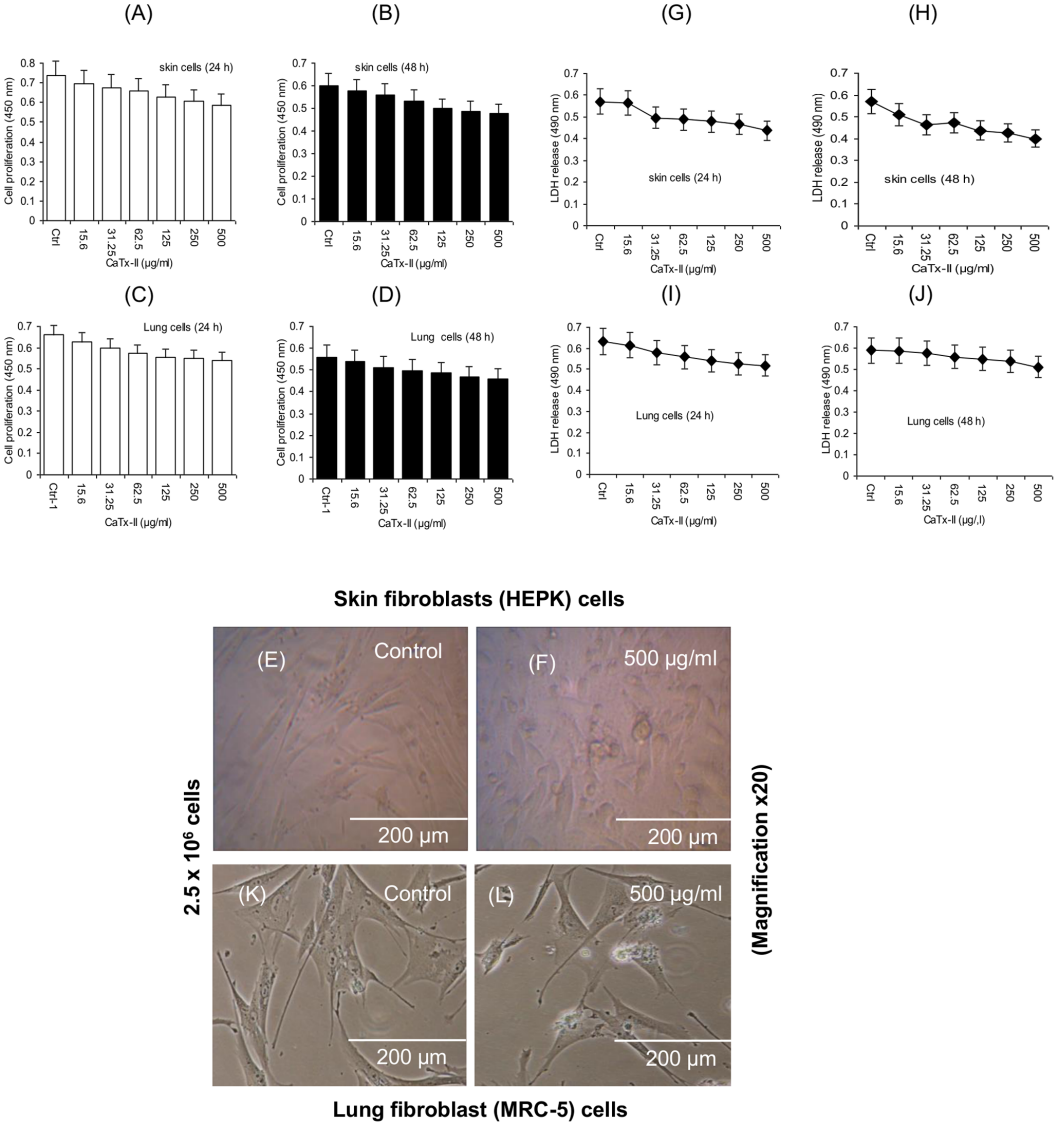
In vitro cytotoxicity was evaluated by XTT-assay. (A–D) CaTx-II incubated with human lung (MRC-5) and skin fibroblast (HEPK) cells using different concentrations (15.6, 31.25, 62.5, 125, 250, and 500 µg/ml) after 24 h and 48 h incubation. The values are expressed as mean ± SD of three replicates (n = 3), Control (Ctrl) cells without any treatment served as a control, (E) Light micrograph showing the normal architecture of skin fibroblasts, (F) cells exposed to CaTx-II at different concentrations 15.6–500 µg/ml. (K) Lung fibroblasts without any treatment, (L) cells treated with 500 µg/ml concentrations of CaTx-II did not cause morphological changes after exposure (Magnification×20), (G–J) Quantification of cell death and lysis was based on lactate dehydrogenase (LDH) activity released from the cytosol of damaged cells into the culture supernatant of the fibroblast cells (skin and lung) exposed to protein up to 15.6–500 µg/ml concentrations. Cells exposed to CaTx-II at 500 µg/ml showed very low level of cytolysis after 48 h exposure.

Cytotoxic effects of CaTx-II were examined on human lung and skin fibroblast cells. Cell viability was not affected even at higher concentrations of 500 µg/ml (Fig. 3A–D). There were no morphological changes, indicating that the cells remain intact with no swelling or death evidenced at higher (up to 500 µg/ml) concentrations of CaTx-II in a time-dependent manner on skin (Fig. 3E–F) and lung (Fig. 3K–L).

### Release of LDH from Cells

LDH results revealed that exposure of the lung and skin fibroblast cells to CaTx-II (up to 500 µg/ml) were not cytotoxic up to 48 h (Fig. 3G–J). However, a higher dose (500 µg/ml) of CaTx-II did not cause LDH release and induced cell death or lysis. However, the growth of fibroblast cells was not affected, especially at the treatment dose up to 500 µg/ml.

### Toxicity Study in Mice

The CaTx-II did not show toxic effects or kill mice up to 14 mg/kg, b.w for 2 weeks, and in fact they behaved like controls given saline only.

### Wound Healing

**Figure 4:**
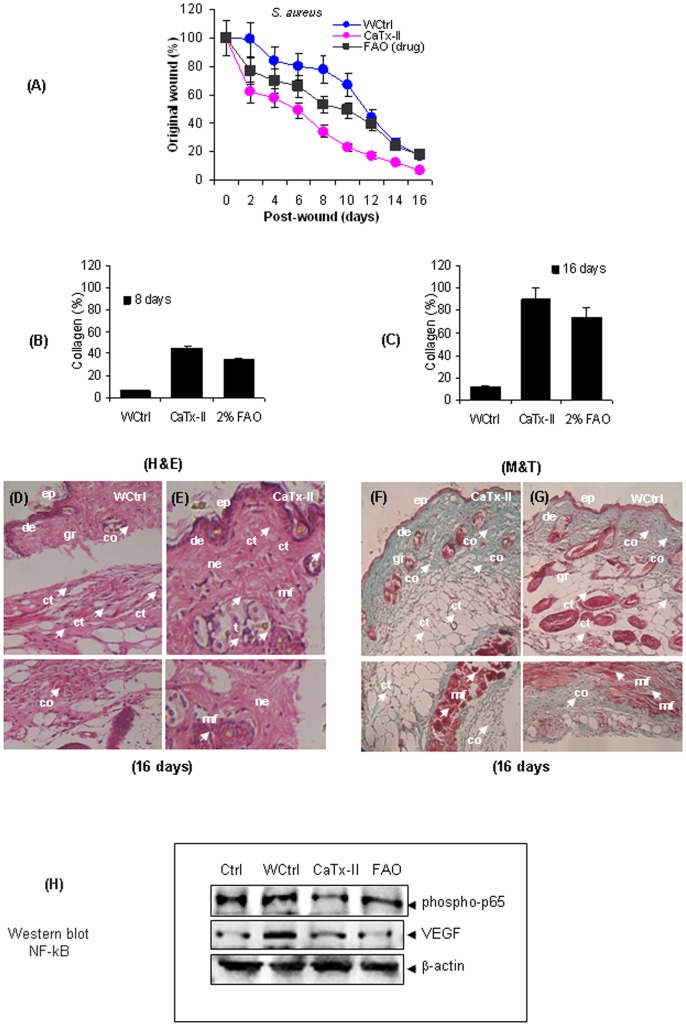
Wound healing is accelerated in CaTx-II treated mice than wound control (WCtrl). (A–B) full thickness excision wounds 2 cm (4 mm×5 mm) were created on the dorsal skin of mice after infected with *S. aureus* (50 µl of 1×10^5^ cfu/ml of bacteria). Wounds were treated by topical application of CaTx-II (10 mg/kg, body weight) or FAO (2%), and wound closure measured and compared with saline-treated control mice (n = 6, *P<0.05). The protein treatment influenced wound healing after 16 days in mice. **Collagen content in wound sites of treated and control animals were measured by ELISA.** (C) There was a significant difference between (CaTx-II) treated animals showing that collagen levels increased to 50% after 8 days versus controls treated with FAO, (D) the collagen content increased to high levels after 16 days of injury in mice. Histopathological examination of skin wound samples of control and protein (CaTx-II) treated mice at 16 days after injury. The section stained with H & E using original magnification (20 x). (E) Wound control mice delayed healing (F) Protein (CaTx-II) treated mice showed significant reduction of wound area, leukocyte infiltration, re-epithelialization, angiogenesis on 16 days after the injury. Masson’s Trichrome (MT) staining of skin wound samples of control and protein (CaTx-II) treated mice at 16 days after injury. (G) CaTx-II treated mice showed excellent accumulation of collagen on mice skin after 16 days post injury, (H) saline received mice showed less stain content of collagen deposition. (I) *S. aureus* bacteria infected skin wounds were either untreated or treated with 10 mg/kg, body weight, of CaTx-II for 16 days. CaTx-II inhibits NF-κB activation and the expression of VEGF in skin wound tissues. Skin wound tissue extracts were prepared and western blot analysis was performed using phospho-specific p65, VEGF and β-actin antibodies.

CaTx-II treated mice showed strong wound healing activity following *S. aureus* infection with a significant reduction in the wound area (60%) after 8 days, compared to that of wound control mice (P<0.01). In contrast, the antibiotic fusidic acid ointment (FAO) treated mice showed only 40% reduction of wound size after 8 days. The CaTx-II treated wounds contracted faster and were completely healed by 16 days as compared with that of the FAO-treated mice. We provide definitive evidence that CaTx-II influences crucial roles in the wound-healing process by regulating leukocyte infiltration, angiogenesis, neovascularization and collagen deposition (Fig. 4 A).

### Collagen Content in Wound Site

The collagen content in wound sites was measured by ELISA. The CaTx-II treated animals showed significantly increased levels of collagen content (p<0.05) as compared with the wound control (WCtrl) animals. CaTx-II treated mice showed a 50% increase in collagen levels after 8 days versus wound control or FAO treated mice. At 16 days post-treatment, the collagen content was increased to 80% in the CaTx-II treated group (Fig. 4 B–C), and 70% in FAO treated group as compared to that of wound control mice.

### Histological Re-epithelialization

Histopathological examinations were carried out on the wounds of treated and untreated mice after 16 days. When compared to the CaTx-II treated wound, the untreated wound epidermis was thin and disorganized; indicating delayed healing and its non-involvement in the regeneration and wound healing process (Fig. 4 D). Also, the significant reduction of infiltrated inflammatory cells, an increase in the formation of blood vessels, and enhanced proliferation of cells were observed in CaTx-II treated wounds. There was thick epidermal regeneration that covered completely the wound area with well-formed granular layers. In addition, there was extensive epithelialization, vascularisation, and hair follicle formation in CaTx-II treated mice. The early dermal and epidermal regeneration in treated mice also confirmed that CaTx-II had a positive effect upon cellular proliferation, granular tissue formation and epithelialization (Fig. 4 E). Masson’s Trichrome staining of control and CaTx-II treated mice after 16 days also evidently supported the collagen accumulation and its expression, which is important for wound healing. CaTx-II treated mice showed a significant accumulation of collagen on mice skin 16 days after the injury (Fig. 4 F), whereas saline injected mice showed very little deposition of collagen (Fig. 4 G).

### CaTx-II Inhibits NF-κB Activation in Skin Wound Model

As NF-κB activation plays a major role in mediating and modulating gene responses associated with wound healing [38], the ability of CaTx-II to modulate NF-κB activation in skin wound model was also investigated. We found that the treatment with CaTx-II substantially inhibited constitutive p65 phosphorylation that was observed in wound control mice (Fig. 4 H). Also, the levels of NF-κB-regulated protein, VEGF, an endothelial cell specific mitogen that plays an important role in vascular development and wound healing [54] was also suppressed upon treatment of mice with CaTx-II (Fig. 4 H).

### Bacterial Counting and Positive Cells at Wound Site

**Figure 5:**
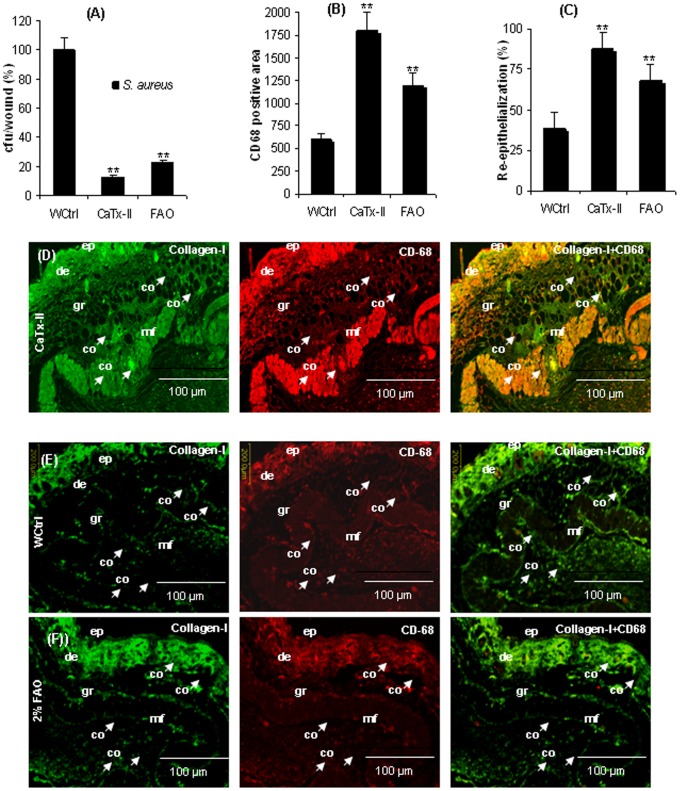
Wound fluid killing assay and determination of positive cells. (A) Wound tissues of treated and untreated mice were collected for bacterial counting. Values are shown as percentage of CFU/wound of three observations. (B) There was very high level of positive cells (CD68 marker for keratinocytes) expressed in CaTx-II treated wound. (C) Representative data of six mice in each group were shown, vascular densities within the wound bed at 8 and 16 days after the injury, with and without treatment, were determined using PhotoShop. All the values represent mean ± SD. **P<0.01, *P<0.05 compared with control. **Immunohistochemical sections of CaTx-II treated, FAO treated, and untreated skin wound samples after 16 days injury.** (D) The section of CaTx-II treated wound stained with collagen (green), stained with CD-68 antigen, merged or co-localization of collagen plus CD-68 (original magnification, ×20). Section of wound control mice skin stained with (E) collagen (green), CD-68 antigen, merge of collagen plus CD-68. (F) Section of wound FAO treated mice skin stained with collagen (green), CD-68 antigen, and merge of collagen plus CD-68. (Magnification bars 100 µm).

Wound tissues of treated and untreated mice were collected for determining the bacterial load as a percentage of CFU/wound of three observations (Fig. 5 A). Representative data of six mice in each group were shown. Vascular densities (re-epithelialization) within the wound bed at 16 days after the injury of CaTx-II or FAO treated mice and untreated mice with injured skin (control) were determined using PhotoShop. All the values represent mean ± SD (n = 6 animals); significance at *P<0.01, **P<0.05 compared with untreated mice wound served as a control.

### Immunohistochemical Examination

Significant bacterial reduction (cfu/wound) was found in wound treated with CaTx-II versus FAO treated and wound control mice (Fig. 5 A). There was a very high level of positive cells (CD68 marker for keratinocytes) expressed in the protein treated wound (Fig. 5 B), and accelerated re-epithelializations (Fig. 5 C) compared with untreated control. Immunostained wound samples of CaTx-II or FAO treated and wound control mice after 16 days are shown in Fig. 5 D–F. The sections were stained with collagen (green), CD-68 antigen (red), or co-localization of collagen plus CD-68. Wound sections of mouse skin topically applied with CaTx-II were treated mice skin intensively stained with collagen (green), CD-68 antigen (red), and a merger of collagen plus CD-68, indicating an interesting over-expression of collagen as well as CD-68 antigen maker for keratinocytes as compared to the WCtrl. The CaTx-II treated wounds expressed or up-regulated more CD68 positive cells and migration of keratinocytes at the wound edge than the other groups (Fig. 5 A–C). The FAO treated section showed less intense staining of collagen and CD-68 antigen than the treated groups.

### Expression of Inflammatory Cytokines

**Figure 6:**
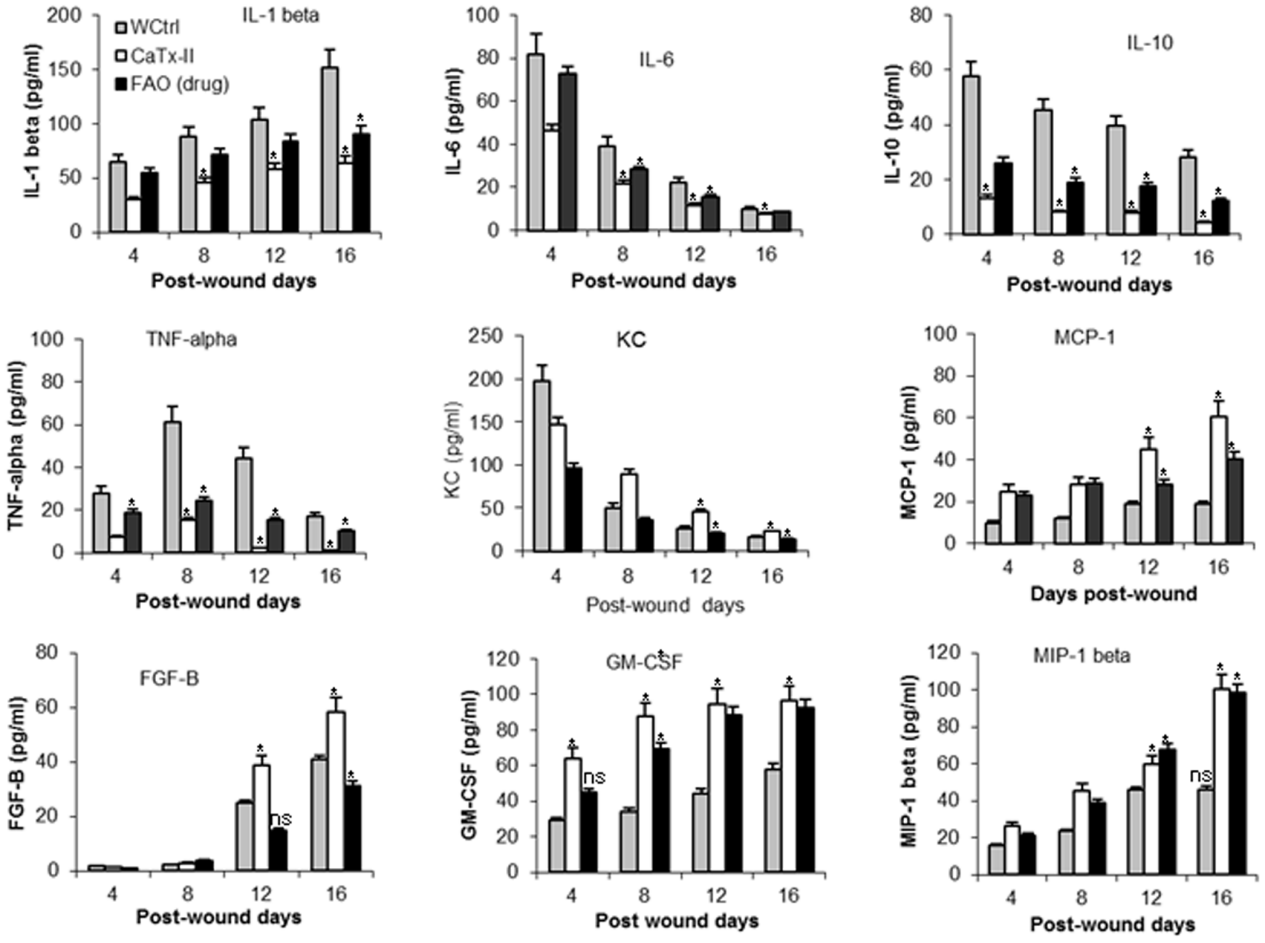
Wound cytokine and chemokine levels as determined by an ELISA. Cytokines were quantified from skin wounds treated with CaTx-II or FAO, compared with untreated wound controls 4–16 days post injury. Cytokine and chemokine levels in skin wound were determined as described in the experimental procedures. Data are mean ± SD, n = 6 mice per group. Statistical analysis was carried out by ANOVA for comparison of multiple groups, levels of significance *P<0.01 as compared with the WCtrl group.

Levels of interleukin 1beta (IL-1β), IL-6, IL-10, TNF-alpha, macrophage inflammatory protein 1 beta (MIP 1 beta), MCP)-1, FGF-Basic, KC, and granulocyte colony-stimulating factor (G-CSF) were measured at 16 days post-treatment in the wound tissue homogenates. With the exception of IL-10, all cytokines measured were elevated significantly versus the control group (Fig. 6). Wounds from both CaTx-II and FAO treated groups contained significantly low levels of the proinflammatory cytokines TNF-alpha, IL-1 and IL-6 versus wound controls. The basal layer keratinocytes at the wound edge showed up-regulated expression of MIP-1 beta. As a result of this mediator increase, the inflammatory response to injury might potentially disrupt other essential phases of wound repair. The reduction in cytokine content ranged from approximately 75% for TNF-alpha, IL-1, IL-6 and IL-10, with no differences in the wound content of MCP-1 measured between the groups. MCP-1 was expressed exclusively around 7 days post-injury, whereas FGF-Basic, KC, and G-CSF were expressed late (16 days) during the recovery phase. FGF basic and G-CSF play an important role in wound healing as components of the cytokine network that regulate co-operation of development and differentiation of cells during repair.

## Discussion

Snake venom phospholipase A_2_ (svPLA_2_s) enzymes are classified into 15 groups and 23 sub-groups. Most of the elapidae and hydrophidae svPLA_2_s belong to group I, whereas viperidae venoms belong to group II. Previously, a number of PLA_2_s have been isolated from snake venoms [1], [40]. In this study, we purified a basic PLA_2_ (CaTx-II) from the eastern diamondback rattlesnake (*C. adamanteus*). Its N-terminal sequence exhibits a high degree (80%) of homology with crotapotin, the Lys-49 PLA_2_ from *C. adamanteus* venom. PLA_2_ homologues from *Crotalus* snake venoms contain 120–130 amino acids with seven disulfide bridges [41]. They are calcium-dependent enzymes that hydrolyze fatty acids at the sn-2 position of phospholipids [42], consisting of catalytically active and inactive forms [43]. The catalytic activity of PLA_2_s depends on the binding of a calcium ion and residue Asp-49 [44]. However, the side-chain atom of lysine residues can play a similar role to that of calcium, thereby enabling Lys-49 PLA_2_ to display interesting catalytic activity [45]. These two types of PLA_2_s exert most of their biological properties through relatively large molecular surfaces encompassing several discrete domains such as the N-terminal helix and calcium-loop [46].

### Antimicrobial Activity

Several antimicrobial proteins and peptides have been identified from both amphibians [47] and reptiles. These molecules are attractive in research due to their broad spectrum of activity on bacteria, viruses and fungi, as well as wound healing effects [1], [24], [25]. *C. adamanteus* venoms are broadly active against Gram-negative and Gram-positive bacteria [48]. The inhibitory and bactericidal activity of *C. adamanteus* venom determined against aerobic and anaerobic microorganisms showed variable activity at 5–20 µg/ml and 480 µg/ml, respectively [49]. This venom was reported to be equally effective as some conventional antibiotics like chloramphenicol and ceftazidime [47], and the bactericidal *action might be due mainly to the enzymatic activity of* C. adamanteus *venom* PLA_2_. In this study, we report for the first time the broad-spectrum antimicrobial activity of CaTx-II purified from *C. adamanateus*e venom. This PLA_2_ was most effective against a Gram-positive (*S. aureus)* and Gram-negative (*B. pseudomallii* strain KHW), with an antimicrobial potency similar to that of antibiotics. CaTx-II was the most active, showing inhibitory activity even at low concentrations of CaTx-II (20 µg) as compared with 30 µg for chloramphenicol and streptomycin.

CaTx-II showed a significant inhibitory effect against *S. aureus* and *B. pseudomallei* strains even at lower concentrations (7.8 and 15.6 µg/ml). These findings are, in contrast with several studies involving bactericidal activity of snake venom PLA_2_ enzymes [16], [17]. Whereas, diverse PLA_2_ enzyme purified from snake venom possessed highly potent antimicrobial activity against multi-drug resistant B. pseudomallei [*15*]. *The reported activity is not only due to a PLA_2_, but also the* basic Asp-49 PLA_2_ subunit of *C. durissus terrificus* venom and displays bactericidal activity upon *E. coli* [22]. In this current study, CaTx-II had minimal effect upon *E. coli*. In addition, myotoxic Asp-49 PLA_2_s and Lys-49 PLA_2_ homologues are potent microbicidal components in snake venoms [74].

The ultrastructural assessment of CaTx-II bactericidal effects, as reported herein, on Gram-positive (*S. aureus* and *E. aerogenes),* and Gram-negative (*B. pseudomalleii*) bacteria suggest that CaTx-II ruptures the membrane subsequently and causes cytoplasm loss. These results clearly provide evidence that CaTx-II acts via its pore-forming action at 7.8 and 15.6 µg/ml doses. It was previously reported that the peptide derived from the C-terminus (115–129) of Lys49 *Bothrops asper* PLA_2_ reproduced bactericidal activity against both Gram-negative (*Salmonella typhimurium)* and Gram-positive (*S. aureus)* bacteria as effectively as its parent molecule [50], [51]. Besides, the bactericidal effect of Lys49 or Asp49 PLA_2_ has been previously reported with the specific membrane-damaging effects [52], [53]. Most Lys-49 PLA_2_s contain a high level of basic and hydrophobic amino acids residues located within the C-terminal region [54]. PLA_2_ homologues bind with high affinity to bacterial lipopolysaccharide (LPS) to block their effects on macrophage and other targets [51]. An earlier study has shown that the PLA_2_-derived peptide interacts with LPS and specifically the lipid A component from diverse Gram-negative bacteria or with lipoteichoic acid from *S. aureus*, and relies on a membrane-permeabilizing mechanism to exert its bactericidal effects [51]. LPS is a complex molecule composed of a fatty acid (lipid A), O-polysaccharide chain and core sugar, inserted into the outer membrane of Gram-negative bacteria. While Lys interacts with the anionic phospholipid head groups in the bacterial membrane surface, the hydrophobic core portion of the peptide can interact with the lipid bilayer causing membrane disruption [55]. Based on our previous studies, most snake venom proteins tested for antibacterial properties induce pore formations (i.e. blebs), likely resulting in loss of membrane integrity and release of cellular contents [1], [31].

Several studies reported that the mechanism action of basic Asp49-PLA_2_ isolated from *Agkistrodon halys* venom induced morphological changes in Gram-negative and positive bacteria consistent with severe damage that include significant membrane blebbing and loss of membrane integrity [31]. The damage was evidenced in *E. coli* by bothropstoxin-1 (Lys49-PLA_2_) isolated of *B. jararacussu*. Lys49-PLA_2_s damage artificial membranes by a Ca^2+^-independent mechanism and displayed a potent bactericidal effect [56]. Bactericidal activity of PLA_2_ involves both recognition of anionic sites and the enzymatic degradation of phospholipids in the membranes of target cells, preferentially of Gram-positive species [57].

Whereas, acidic PLA_2_ of *Porthidium nasutum* caused morphological changes consisting of irregular-shaped bacterial cell wall, cellular debris, and possible leakage due to cell lysis (Vargas et al., 2012). It well recognized that hydrolysis of the phospholipid component of the bacterial cell membrane by PLAs is involved in killing both Gram-positive and Gram-negative bacteria. PLA2 are able to penetrate the peptidoglycan envelope of Gram-positive bacteria and gain access to the cell membrane phospholipids. However, they are capable of hydrolyzing the phospholipids of the bacterial cell membrane in Gram-negatives only after the lipopolysaccharide-rich layer is disrupted. Thus the effects on these bacteria depend on factors that can disrupt the outer membrane [58].

### *In vitro/in vivo* Toxicity

CaTx-II did affect viability of human lung (MRC-5) and skin fibroblast (HEPK) cells in a dose- and time-dependent manner. *In vitro* assay showed that after 24 and 48 h of treatment, metabolism was not inhibited at a very high dose of CaTx-II (2000 µg/ml). *In vivo*, the CaTx-II (1–14 µg/kg, b.w) did not show toxic effects or kill mice after 2 weeks, and in fact they behaved like controls. A previous study showed that the neutral PLA_2_ from *Hemachatus haemachatus* venom is much less toxic in mice than the basic PLA_2_ from *Naja nigricollis* venom, with an i.v LD_50_ of 8.6 and 0.63 mg/kg, respectively [59]. Similarly the injection of neutral PLA_2_ into rats showed convulsant and lethality effects at 7.5 and 15 µg, respectively. Corresponding values for the basic PLA_2_ were quite lower at 0.5 µg per rat. Death appeared due to congestion, hemorrhage and edema in the lungs. A direct effect on the cardiac or respiratory system depends upon the route of administration [54].

### Wound Healing

Wound healing is a complex process involving a series of overlapping, inflammation-based phases [60]. White blood cells invade the wound region by migrating through the extracellular matrix (ECM). Fibroblasts subsequently migrate into the injured site and replace the blood clot with collagen. These latter cells biochemically alter the ECM by degrading the fibrin and producing collagen [61]. In mice, when CaTx-II was topically applied to a wound after *S. aureus* infection, healing was significantly accelerated as a result via the contraction of newly formed connective (granulation) tissue by fibroblasts. Our results corroborate that recently reporting [61] the formation of connective tissue by fibroblasts during wound healing. During the final phase of wound healing (maturation phase), there is contraction, new tissue formation resulting in a smaller amount of apparent scar tissue [35]. In addition, protein-based antimicrobials like CaTx-II can be multifunctional with receptor-mediated effects on eukaryotic cells that include chemotaxis and wound healing [2], [3], [62]. Differential expression of antimicrobial proteins/peptides plays an important role in the susceptibility of patients with inflammatory skin disorders to infections. In addition, the neutrophils are abundant in lesions of superficial skin disease characterized by inflammation and infection with *S. aureus* [63]. These studies indicate potential roles for antimicrobial peptides in host immune defense against skin infection.

Interestingly, CaTx-II treatment influences the migration and proliferation of keratinocytes at the wound edge followed by proliferation of fibroblasts into the adjacent area. The treated animals also experienced accelerated neovascularization, granulation tissue formation, and re-epithelialization of the wound as a result of increased keratinocyte proliferation. Wound healing is firmly-based upon collagen. Proliferation of fibroblasts and synthesis of new collagen predominate in the proliferative phase of the healing process [64]. The quantitative assessment of collagen deposition rates of both CaTx-II treated and control groups used for the measurement of wound healing responses after 16 days. CaTx-II treatment leads to better wound healing versus untreated. Immunohistochemistry studies also indicated that type I collagen is the most abundant protein component of the granulation tissue and accumulated more than the surrounding dermis. Detection of the expression and distribution of CD68 (the surface marker of macrophages) revealed a more prevalent pattern in wounds treated with CaTx-II, versus untreated, after 16 days. Previously, anti-CD68 antibody was used to study the role of haemoxygenase-1 in wound repair [65].

### Inflammatory Cytokines

Inflammatory cells invade the wound tissue within a few hours after injury [66], and these cells are an important source of growth factors and cytokines that initiate the proliferative phase of wound healing. All stages of the wound healing process are controlled by a wide variety of different growth factors, chemokines and cytokines [67]. Particularly, the cytokines influence various processes at the wound site such as stimulation of keratinocyte, granulation tissue formation [55], fibroblast proliferation, synthesis of collagen, and breakdown of ECM. Several cytokines such as IL-1 alpha, IL-1beta, IL-6 and TNF-alpha play a major role in wound healing. Expression of IL-1 beta, IL-6 and TNF-alpha is strongly up-regulated during the inflammatory phase of healing [66], [68]. In this study, the wound control group contained significant elevated levels of all cytokines with the exception of IL-10 in mice. Wounds treated with either CaTx-II or FAO showed significantly low levels of inflammatory cytokines such as TNF-alpha, IL-1 and IL-6 compared with the WCtl. Previously, Mori et al [69] generated excisional wounds on the back of mice lacking the TNF receptor p55 and showed more collagen content, angiogenesis and re-epithelialization. In this current study, the expression of TNF-alpha was also increased from the early phase of the wound at 4 days. Whereas, the wound control (WCtrl) had up-regulated expression of MIP-1, and as a result of this mediator there was an increased inflammatory response to injury that potentially disrupted other essential phases of wound healing. At the wound site, CaTx-II enhanced expression of KC, MCP-1, FGF-basic and G-CSF after 16 days. In addition to leukocyte infiltration, expression of adhesion molecules and cytokines were reduced significantly. FGF basic and GM-CSF play important roles in wound healing as components of the cytokine network that regulates development and differentiation of cells during wound healing. Fascinatingly, various growth factors essential for wound healing, such as TGF-β1, are elevated in the wounds of animals [70]. Histological data were also supported by increased expression of TGF-beta 1 at the wound site [64]. However, studies involving full-thickness excisional wounds generated in transgenic mice over expressing GM-CSF in the epidermis [70] show that GM-CSF stimulates wound healing directly. This molecule regulates growth/differentiation of various cells that include keratinocytes and endothelial cells. Keratinocytes actively control the production of their growth factors in fibroblasts by release of interleukin-1 (IL-1) alpha and beta, enhancing expression of growth factors in stromal cells such as keratinocyte growth factor (KGF) and fibroblast growth factor-7 (FGF-7), and granulocyte-macrophage colony-stimulating factor (GM-CSF). These factors strongly stimulate keratinocyte proliferation and are up-regulated during wound healing [71].

NF-κB is a heterodimeric transcription factor that plays a key role in inflammatory and immune response [72]. Transcription of various inflammatory cytokines and CXC chemokines requires the activation of NF-κB, a major target of IL-1 and TNF-alpha mediated signal pathway [39]. These cytokines (IL-1 and TNF-alpha) can induce the phosphorylation and subsequent degradation of IκB, followed by liberation of NF-κB and its nuclear translocation. In the nucleus, NF-kB dimers bind to target DNA elements and activate transcription of immune and/or inflammatory-associated genes, such as chemokines and adhesion molecules [73]. Recently, several lines of evidence indicate that the transcription of VEGF, a potent angiogenic factor, is also regulated in a TGF-beta/Smads-dependent manner [74]. TGF-beta 1 is implicated as a key mediator of collagen synthesis [75]. It activates the kinase activities of its receptor, which phosphorylated Smad2 and Smad3 associate with Smad4 and translocate into nucleus, thereby inducing expression of the target genes. In this study, the ability of CaTx-II protein to modulate NF-κB activation in a skin wound tissues was also investigated. We found that the treatment with CaTx-II substantially inhibited constitutive p65 phosphorylation that was observed in wound control mice. Also, the levels of a NF-κB-regulated protein, VEGF [76] were also substantially suppressed upon treatment of mice with CaTx-II. VEGF enhances the permeability of local blood vessels [77] and recently, Peters et al [78] has previously demonstrated substantial expression of VEGF mRNA in proliferating keratinocytes of the newly formed epithelium during wound healing.

## Conclusions

CaTx-II possesses significant sequence similarity with the Lys-49 PLA_2_ enzymes. This protein is effective as an antimicrobial against Gram-positive *S. aureus* bacteria and Gram-negative *B. pseudomallei and E. aerogenes* bacteria versus tested antibiotics. Our results provide an excitedly new insight upon the antimicrobial mechanism of a membrane damaging snake venom protein that includes pore-formation. CaTx-II did not have cytotoxicity on tested human lung and skin fibroblast cells at high doses. On the other hand, CaTx-II promotes significantly better wound healing activity than fusidic acid (FAO) ointment. CaTx-II accelerates wound healing, faster re-epithelialization, formation of collagen, as well as enhanced levels of MCP-1, FGF-basic, KC, and G-CSF after 16 days. Whereas, the wound treated with CaTx-II significantly inhibited p65 phosphorylation and expression compared to the wound control. In addition, the levels of NF-κB-regulated protein, VEGF an endothelial cell specific mitogen that plays an important role in vascular development and wound healing in this study. The clinical significance of CaTx-II may be its utility as a potent wound healing agent for the treatment of skin wounds. The rapid development of antibiotic resistant bacteria, with fewer efficacious options available to the physician, makes it necessary to discover novel antimicrobial agents. Our findings suggest that snake venoms represent a potentially widespread source of novel antimicrobial agents that may have important medical applications in the near, and distant, future.

## Supporting Information

Figure S1 **Enzyme linked immunoabsorbent assay (ELISA) based biochemical analysis.** (A–B) PLA_2_ activity was compared with the *C. adamanteus* crude venom (CA-CV), different purified fractions (CA-F1-CA-F4), and CaTx-II. (C–D) Total protein contents were determination. Values are mean ± SD of three replicates, the highest PLA_2_ activity containing fraction is indicated by asterisks.
(TIF)

Figure S2 ***In vitro*** **antimicrobial activity of CaTx-II determined by a standard zone of inhibition assay against Gram-positive and Gram-negative bacteria.** (A–C) The purified protein CaTx-II displayed the most potent growth inhibitory (millimeter in diameter) potential against *B. pseudomallei, S. aureus* and *E. aerogenes* than the separated fraction (CA-F1) and crude venom. (CA-CV). Clear zone of inhibition measured around the disc after 24 h incubation at 37°C. (D–E) The antimicrobial potency of CaTx-II was almost equal when compared to that of commercial antibiotics such as CHL-chloramphenicol and Stp-stretomycin against tested bacteria.
(TIF)

Figure S3 **Bacteriostatic effect of CaTx-II was determined by MH and TS broth dilution method at different doses (3.9–250 µg/ml) incubated with bacteria (10^5^–10^6^ CFU/ml) for 24 h at 37°C.** Bacterial turbidity was measured spectrophotometrically at 560 nm. Each optical density (OD) values represents the mean ± SD of three replicates (n = 3). The MIC values represented by (#) of each Gram-positive and Gram-negative bacteria (A) *B. pseudomallei* KHW (MIC of 7.8 µg/ml), (B) *B. pseudomallei* TES (MIC of 15.6 µg/ml), (C) *S. aureus* (MIC of 7.8 µg/ml), (D) *E. aerogenes* (MIC of 31.25 µg/ml), (E) *P. vulgaris* (MIC of 31.25 µg/ml), (F) *P. mirabilis* (MIC of 62.5 µg/ml), (G) *K. pneumoniae* (MIC of 62.5 µg/ml), (H) *S. pneumoniae* (MIC of 250 µg/ml), (I) *P. aerogenes* (MIC of 125 µg/ml).
(TIF)

Figure S4 ***In vitro*** **bactericidal activity of CaTx-II on bacteria.** Different concentrations of protein (3.9–250 µg/ml) were serially diluted with MH and TS broth containing (10^5^–10^6^ CFU/ml). 50 µl of this sample was added to 96-well plates and incubated at 37°C for 24 h. After incubation, 20 µl of the sample was applied onto MH and TS agar plates and viable counts of the bacteria recorded after 24 h. The CFU of each point represents mean ± SD of triplicate counts (n = 3).
(TIF)

Table S1 The N-terminal amino acid sequences of purified PLA_2_ protein (CaTx-II) from *Crotalus adamanteus* was compared with other snake venom PLA_2_s such as PA_2_A_CRODU-Crotoxin acid chain (*Crotalus durissus terrificus*), PA2A_CROSS-Mojave toxin acidic chain (*Crotalus scutulatus scutulatus*), PA21B_TRIGA-PLA_2_ isozyme (*Trimeresurus gramineus*), PA2_CROAT-PLA_2_ (*Crotalus atrox*), PA2C_AGKRHPLA2 (*Agkistrodon rhodostoma*), PA_2__BOTPC-PLA_2_ (Bothrops pictus), PA21_ECHCO-PLA_2_ (*Echis coloratus*), PA2B2_BOTJRbothropstoxin- 2 (*Bothrops jararacussu*), PA25_ECHPL-PLA2 (*Echis pyramidum leakeyi*), PA21_BOTAS-PLA_2_ (*Bothrops asper*).
(DOC)
