# Supplementary figures and images for: Wound Healing Activity and Mechanisms of Action of an Antibacterial Protein from the Venom of the Eastern Diamondback Rattlesnake (Crotalus adamanteus)
Source: PLoS One. 2014 Feb 14;9(2):e80199. doi: 10.1371/journal.pone.0080199 (PMC3925076; doi:10.1371/journal.pone.0080199)

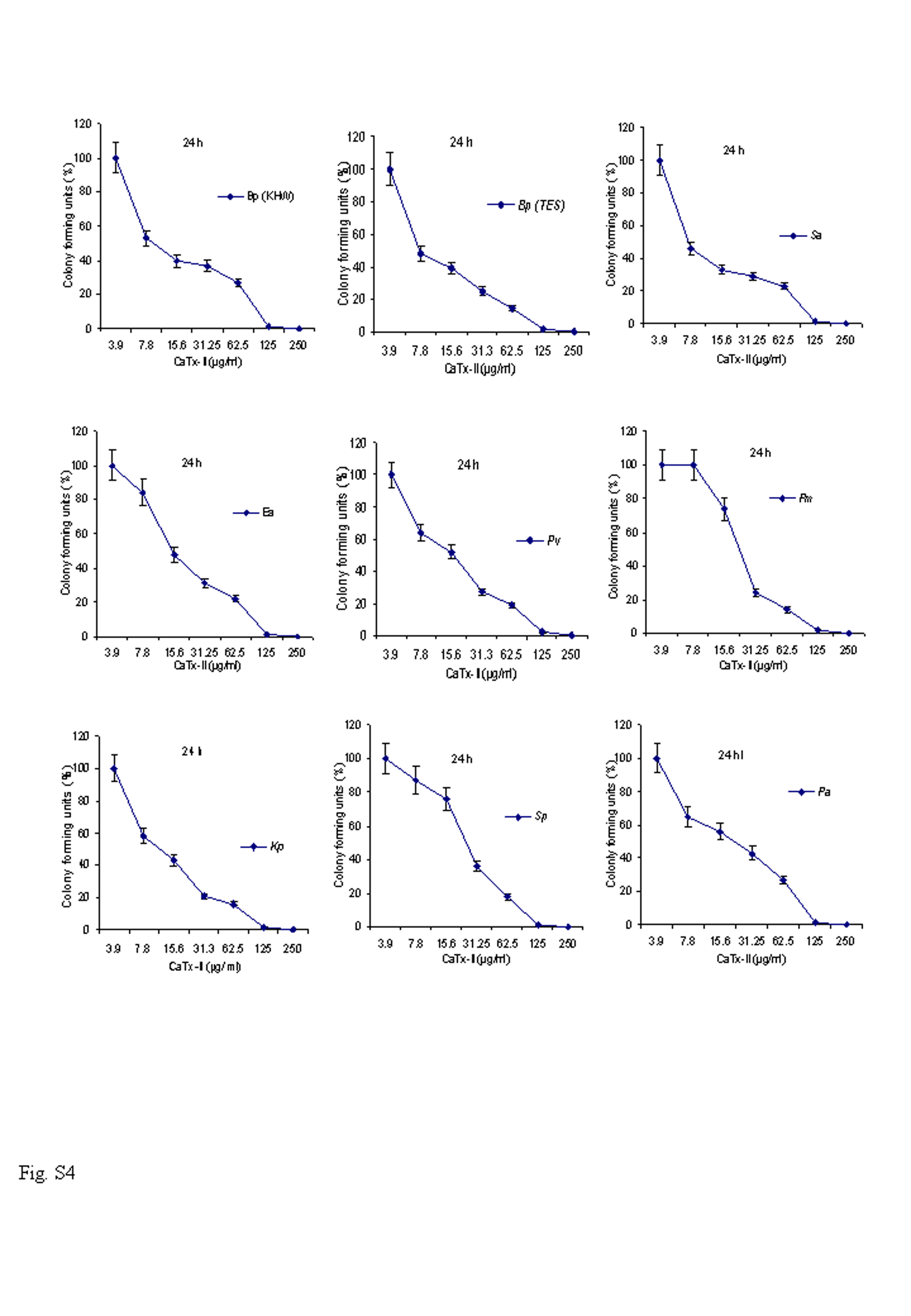

Supplement: Figure S4 — In vitro bactericidal activity of CaTx-II on bacteria. Different concentrations of protein (3.9–250 µg/ml) were serially diluted with MH and TS broth containing (105–106 CFU/ml). 50 µl of this sample was added to 96-well plates and incubated at 37°C for 24 h. After incubation, 20 µl of the sample was applied onto MH and TS agar plates and viable counts of the bacteria recorded after 24 h. The CFU of each point represents mean ± SD of triplicate counts (n = 3). (TIF) [file pone.0080199.s004.tif]
