# Supplementary material for: Wound Healing Activity and Mechanisms of Action of an Antibacterial Protein from the Venom of the Eastern Diamondback Rattlesnake (Crotalus adamanteus)
Source: PLoS One. 2014 Feb 14;9(2):e80199. doi: 10.1371/journal.pone.0080199 (PMC3925076; doi:10.1371/journal.pone.0080199)
Supplement: Table S1 — The N-terminal amino acid sequences of purified PLA2 protein (CaTx-II) from Crotalus adamanteus was compared with other snake venom PLA2s such as PA2A_CRODU-Crotoxin acid chain (Crotalus durissus terrificus), PA2A_CROSS-Mojave toxin acidic chain (Crotalus scutulatus scutulatus), PA21B_TRIGA-PLA2 isozyme (Trimeresurus gramineus), PA2_CROAT-PLA2 (Crotalus atrox), PA2C_AGKRHPLA2 (Agkistrodon rhodostoma), PA2_BOTPC-PLA2 (Bothrops pictus), PA21_ECHCO-PLA2 (Echis coloratus), PA2B2_BOTJRbothropstoxin- 2 (Bothrops jararacussu), PA25_ECHPL-PLA2 (Echis pyramidum leakeyi), PA21_BOTAS-PLA2 (Bothrops asper). (DOC) [file pone.0080199.s005.doc]

**Table S1.** The N-terminal amino acid sequences of purified PLA_2_ protein (CaTx-II) from *Crotalus adamanteus* was compared with other snake venom PLA_2_s such as PA2A_CRODU-Crotoxin acid chain (*Crotalus durissus terrificus*), PA2A_CROSS-Mojave toxin acidic chain (*Crotalus scutulatus scutulatus*), PA21B_TRIGA-PLA_2_ isozyme (*Trimeresurus gramineus*), PA2_CROAT-PLA_2_ (*Crotalus atrox*), PA2C_AGKRH-PLA_2_ (*Agkistrodon rhodostoma*), PA2_BOTPC-PLA_2_ (*Bothrops pictus*), PA21_ECHCO-PLA_2_ (*Echis coloratus*), PA2B2_BOTJR-bothropstoxin-2 (*Bothrops jarara*cussu), PA25_ECHPL-PLA_2_ (*Echis pyramidum* *leakeyi*), PA21_BOTAS-PLA_2_ (*Bothrops asper*). Expert protein analysis system (ExPASy) tool was used for the basic local alignment tool (BLAST) to search for similarity and multiple sequence alignments between PLA_2_ sequences. Completely conserved residues in all sequences are bolded and marked by asterisks. Gaps are inserted into the sequences to attain maximum homology.

| ***Toxin names*** | ***Accession No*** | **Signal sequences N-terminal Ca2+ -loop active site** |
| --- | --- | --- |
| **CaTx-II PA2A_CRODU PA2A_CROSS PA21B_TRIGA PA2_CROAT PA2C_AGKRH PA2_BOTPC PA21_ECHCO PA2B2_BOTJR PA25_ECHPL PA21_BOTAS** | **PLA_2_**  sp|P08878| sp|P18998| sp|P20476| sp|P00624|  sp|Q9PVE9|  sp|Q9I8F8|  sp|Q90ZZ9|  sp|P45881|  sp|P59172| sp|P20474| | **1 10 20 30 40 50**  **----------------SLVQFETLIMKVAKRSGLLWYSAYGCYCGWGGQGWP**-----------MRALWIVAVLLVGVEGSLVEFETLMMKIAGRSGISYYSSYGCYCGAGGQGWPQDASDRCCFEHDCC  MRALWIVAVLLVGVEGSLVEFETLIMKIAGRSGISYYSSYGCYCGAGGQGWPQDASDRCCFEHDCC  MRTLWIMAVLLVGVEGHLMQFETLIMKVAGRSGVWYYGSYGCFCGAGGQGRPQDASDRCCFVHDCC  MRTLWIVAVLLLGVEGSLVQFETLIMKIAGRSGLLWYSAYGCYCGWGGHGLPQDATDRCCFVHDCC  MRTLWILAVLLVGVEGSLVQFETMIMKLAKRSGFFWYSFYGCYCGWGGHGLPQDPTDRCCFVHDCC  ----------------SLVQFETLIMKIAKRSGVWFYGSYGCFCGSGGQGRPQDASDRCCFVHDCC  MRTLWIVAVWLMSVEGNLYQFGKMIKNKTGKPAMFSYSAYGCYCGWGGQGKPQDASDRCCFVHDCC  MRTLWIMAVLLVGVEGDLWQFGQMILKETGKLPFPYYTTYGCYCGWGGQGQPKDATDRCCFVHDCC  MRTLWIVAVWLMGVEGNLYQFGKMIKNKTGKPAMFSYSAYGCYCGWGGQGKPQDPSDRCCFMHDCC  MRTLWIMAVLLVGVEGSLIEFAKMILEETKRLPFPYYTTYGCYCGWGGQGQPKDATDRCCFVHDCC  * :* :: : : : . * ***:** **:* |

CLUSTAL W (1.83) multiple sequence alignment.
